# Does resveratrol improve cognition in humans? A scientometric study to an in‐depth review

**DOI:** 10.1111/cns.14276

**Published:** 2023-05-29

**Authors:** Wenling Tu, Meiying Song, Xiang Fan

**Affiliations:** ^1^ School of Basic Medical Sciences Zhejiang Chinese Medical University Hangzhou China

**Keywords:** Clinical application, cognitive function, resveratrol, scientometric study

## Abstract

**Aim:**

In order to understand the different processes and mechanisms of cognitive function and resveratrol (RES) as an active participant in pathophysiological events that affect cognitive function.

**Methods:**

First, the Web of Science (core collection) was selected as the data source. To ensure the comprehensiveness and accuracy of the search data, the index was selected as “SCI‐EXPANDED”, and the search formula was TS = resveratrol AND TS = (“cognitive” OR “memory” OR “cognition” OR “mood”). Next, details of authors, keywords, journals, countries, institutions, references, and more were analyzed by CiteSpace and VOSviewer software. Finally, we explored the mechanism by which RES could improve cognitive impairment, that involves healthy young adults, healthy elderly, post‐menopausal women, and diseases involving Alzheimer's disease (AD), diabetes‐related cognitive impairment, mental illness, post‐stroke cognitive impairment, and neonatal hypoxic–ischemic injury.

**Results:**

287 valid papers were obtained. The scientometric results demonstrated 287 papers used in this study came from 1601 authors from 443 organizations in 38 countries, published in 169 journals, and cited 13,680 literatures from 2431 journals. Depression, AD, cerebral ischemia, diabetic cognitive impairment, and cognitive function in the elderly are all keywords of the co‐occurrence network.

**Conclusion:**

This study supports the hypothesis that chronic RES intake may positively affect brain function. But it has become challenging to determine the optimal dose, time and duration of RES and improve the bioavailability of RES, which many researchers need to overcome.

## Graphical abstract

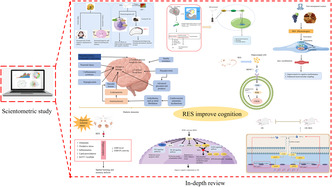

## 1 INTRODUCTION

Cognition is the process by which the human brain tissue receives and processes information, converts information into psychological activities, and then obtains the required knowledge and information, including language, calculation, memory, judgment, and execution. Although sometimes investigators will study each functional region separately, various domains of cognitive function are always interrelated and play a role together.^1^, ^2^ Cognitive impairment refers to the pathological process in which the human body has abnormal intellectual processing processes related to learning, memory and thinking as well as judgment, which causes severe learning and memory impairment, accompanied by changes such as aphasia, apraxia, agnosia, or disturbance in executive function.^3^ There are many factors causing cognitive impairment, of which age is one of the important factors, and the cognitive function of the elderly will inevitably decline with age,^4^ which may also be triggered by a variety of neurodegenerative diseases, vascular cognitive impairment, brain injury and other diseases.^5^, ^6^, ^7^, ^8^

With the deepening of the “silver wave” process, the aging of the population has gradually become a new normal that is difficult to reverse in society and has gradually become a new challenge faced by the whole society, meanwhile, cognitive impairment is an important threat factor in the process of healthy aging of the elderly,^9^ so the search for effective drugs to treat or prevent cognitive impairment is the focus of many researchers. Resveratrol (RES) is a natural non‐flavonoid polyphenol compound that is mainly present in various fruits such as grapes, berries, and peanuts, and it is indicated that RES has a variety of pharmacological effects, including anti‐inflammatory,^10^ antibacterial, antioxidant, neurological and cardiovascular protection.^11^ Furthermore, RES has been found to have a strong anti‐aging effect, enhancing memory and delaying brain function decline,^12^ therefore more and more researchers have investigated whether RES can improve cognitive impairment. It is interesting and meaningful to study the effect of RES treatment on the regulation of cognitive function, there is no scientometric analysis of the regulation of cognitive function by RES has been performed to date.

Scientometrics first emerged in the early 20th century, and in 1969, it formed an independent discipline and was widely used in literature analysis.^13^ Details such as authors, keywords, journals, countries, institutions, and references can be obtained during the analysis. We need to use CiteSpace 6.1.3 and VOSviewer 1.6.18 software in scientometric analysis. CiteSpace can clearly outline the process of knowledge evolution and the historical span of literature in a certain cluster in the time dimension and understand the development process and trends in this field.^14^ VOSviewer uses a probability‐based data normalization method, which provides a variety of visual views in the fields of keywords, co‐institutions, and co‐authors, with prominent characteristics of simple drawings and beautiful images.^15^ Scientometric research can summarize the current hotspots and provide future research directions for a specific field. Therefore, scientometric methods were selected to explore and discover the research hotspots of RES in improving cognitive function in this study.

## 2 SCIENTOMETRIC STUDY

### 2.1 Literature search, screening and download

**FIGURE 1:**
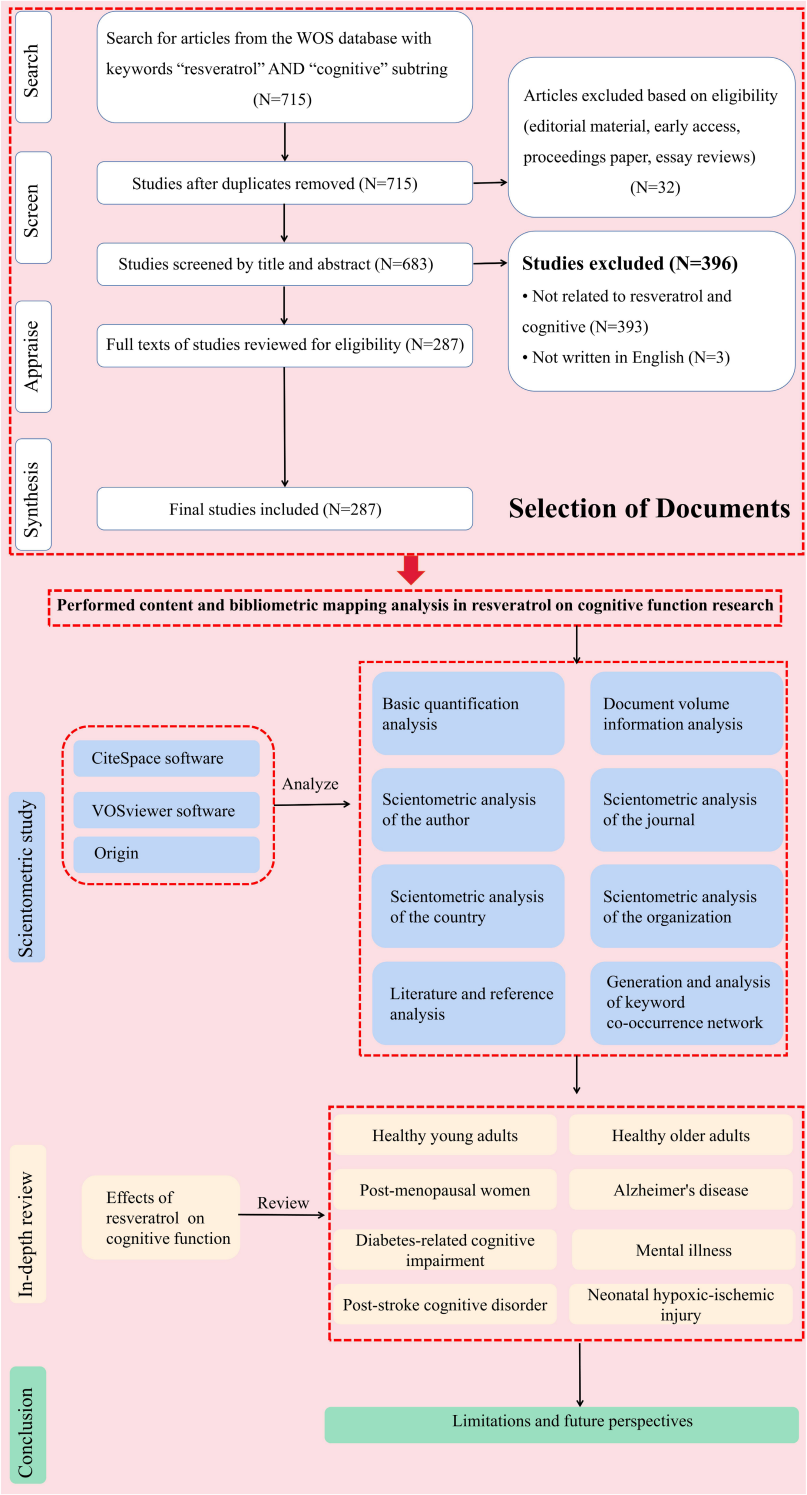
The flow chart of study of RES on cognitive function.

In this study, Web of Science (core collection)^16^ was selected as the data source, and in order to ensure the comprehensiveness and accuracy of the search data, the index was selected as “SCI‐EXPANDED”, and the search formula was TS = resveratrol AND TS = (“cognitive” OR “memory” OR “cognition” OR “mood”).^17^ The time span was from 2008 to 2022. The search deadline was October 25, 2022. A total of 715 articles were obtained. Subsequently, the types of articles were selected as Articles and Review Articles. After the results were reanalyzed, a total of 683 journal articles were obtained. Then the data cleaning was performed on the retrieved papers. Finally, 287 valid papers were obtained. The data cleaning steps are shown in Figure 1.

### 2.2 Basic quantification analysis

The 287 papers used in this study came from 1601 authors from 443 organizations in 38 countries, published in 169 journals, and cited 13,680 literatures from 2431 journals.

### 2.3 Document volume information analysis

**FIGURE 2:**
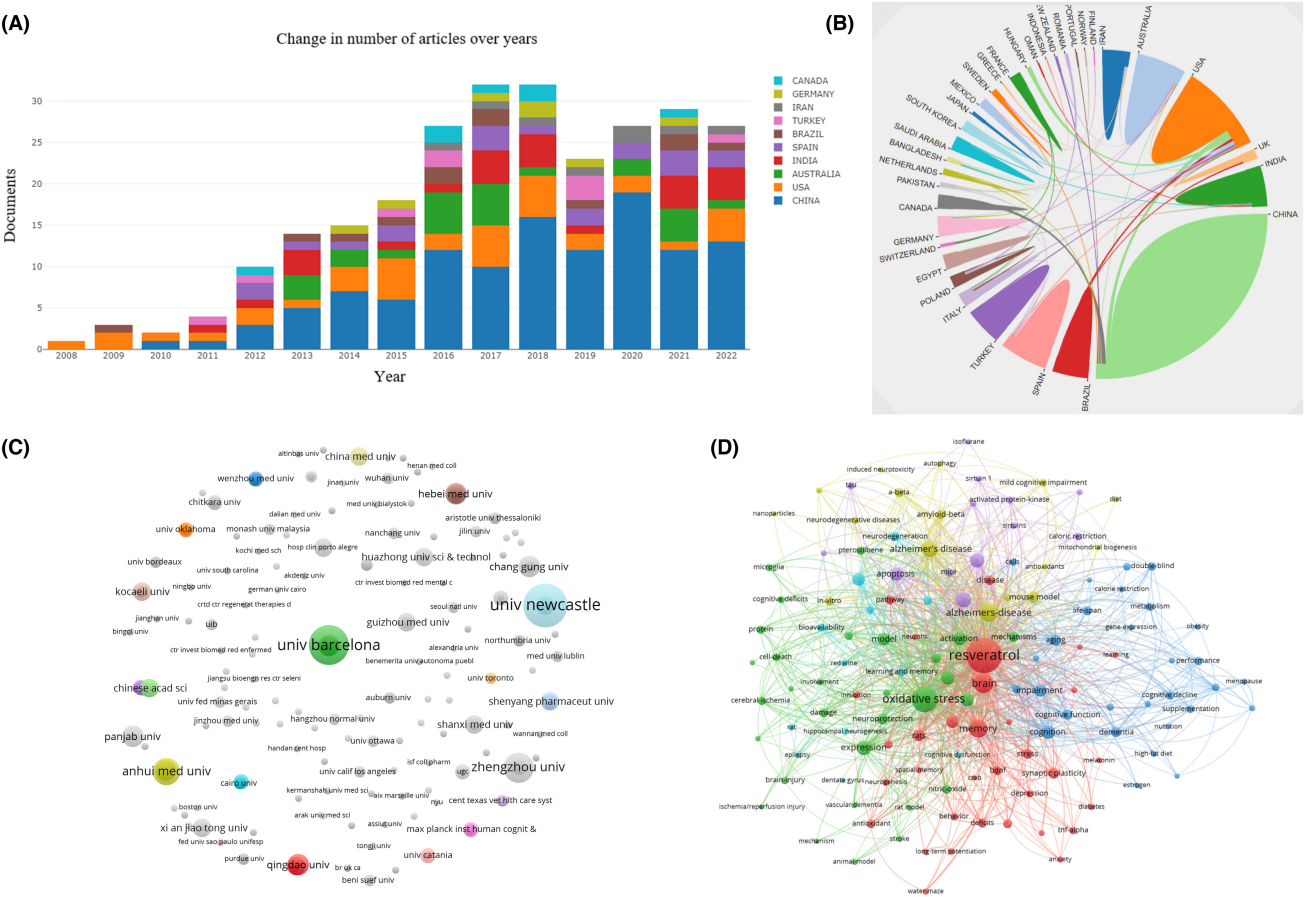
Scientometric study of resveratrol on cognitive impairment. (A) Distribution of publications from 2008 to 2022. (B) The cooperation relationships of countries or regions. (C) Institutions co‐occurrence network. (D) Keyword co‐occurrence diagram from 2008 to 2022.

Figure 2A shows the time of publication of RES articles in cognitive function research. Overall, RES articles related to cognitive function have only gradually emerged since 2008. The growth of publications on this topic can be divided into two stages, 2008–2011 as a slow period and 2011–2022 as a rapid growth period. The rapid growth in research on RES and cognitive function is because increasing evidence in recent years has revealed the effects of the natural product RES on cognitive function, which is currently the focus of numerous researchers.^18^, ^19^, ^20^

### 2.4 Scientometric analysis of the author

By analyzing the authors of the literature, representative scholars and core research forces in this research field can be obtained. Table 1 shows high‐productivity authors who have issued 5 or more documents in this field.

**TABLE 1:** Most important authors in the RES and cognitive function research field.

Rank	Author	Documents	Citations	Average citation/publication
1	Merce Pallas	11	632	57.46
2	Peter r.c Howe	8	339	42.38
2	Rachel h.x Wong	8	339	42.38
4	Coral Sanfeliu	6	436	72.67
5	Christian Grinan‐ferre	6	228	38.00

Among the high‐productivity authors, Merce Pallas published the most documents from 2008 to October 2022, obtaining a total of 11 documents and 632 citations, with the average paper citation times reaching 57.46. Peter r.c Howe and Rachel h.x Wong ranked second, 8 documents and 339 citations were obtained, and 42.38 average citations were obtained; The author with the most average paper citation times was Coral Sanfeliu. Merce Pallas served at Biomed Res Networking Ctr Neurodegenerat Dis CIBER; Coral Sanfeliu served at CIBER‐Centro de Investigacion Biomedica en Red, and these two scholars collaborated several times. Peter r.c Howe and Rachel h.x Wong all served at the University of Southern Queensland and collaborated several times. From the analysis of published papers by these authors, scholars were more concerned about the relationship between RES and aging‐related cognitive impairment, the relationship between RES and cognitive impairment in Alzheimer's disease (AD), the relationship between RES and cognitive function in post‐menopausal women, the relationship between RES and cognitive impairment in diabetes and so on.

### 2.5 Scientometric analysis of the journal

According to the statistics of the journals to which the literature belongs, it is found that in the past decade, most of the journals accepting papers in this field belong to the fields of neurosciences, pharmacology, and biochemistry molecular biology except for a small number of comprehensive journals, and Table 2 shows the journals with the top 10 articles. The journal with the largest number of articles is Brain Research. Frontiers in Neuroscience, Nutrients and Plos One belong to open source journals, indicating that the vigorous development of open source journals in recent years has strongly promoted the research progress in this field. Although scholars still have differences in the best way to achieve open access, the idea that research results should be provided free of charge has been widely recognized. Analysis of the citations of the journals in Table 2 revealed that the journal with the most average citations was “Plos One”, with a total of 5 articles, and the per article were cited up to 62 times, which indicated that the articles published in this journal were of high quality and received much attention in the field of RES and cognitive function.

**TABLE 2:** Top 10 journals in the RES and cognitive function research field.

Rank	Source	Documents	Citations	Average citation/publication
1	Brain Research	7	199	28.43
2	Molecular Neurobiology	6	311	51.83
3	Frontiers in Neuroscience	6	166	27.67
4	Neurochemistry International	6	319	53.17
5	Journal of Nutritional Biochemistry	6	119	19.83
6	Nutrients	6	233	38.83
7	Molecular medicine reports	6	114	19.00
8	Behavioral Brain Research	5	290	58.00
9	Neuroscience	5	197	39.40
10	Plos One	5	310	62.00

### 2.6 Scientometric analysis of the country

In order to understand which countries have contributed most prominently to the field of RES and cognitive function research, this study analyzed the number of documents issued in 38 countries. Table 3 presents the top 10 countries in this field. From the analysis of Table 3 data, it can be seen that Chinese scholars have contributed the most research papers in this field, and a total of 119 papers have been published, accounting for 41.46% of the total number of documents issued in this field, with a total of 3058 citations. Second, the United States, with a total of 37 documents issued, obtained 1999 citations, but also the country with the most average citations. Partnerships between countries showed close cooperation between countries, and the results are shown in Figure 2B.

**TABLE 3:** Top 10 countries in the RES and cognitive function research field.

Rank	Country	Documents	Citations	Average citation/publication
1	Peoples R China	119	3058	25.70
2	USA	37	1999	54.03
3	Australia	24	883	36.79
4	India	24	521	21.71
5	Spain	19	931	49.00
6	Brazil	14	505	36.07
7	Turkey	10	235	23.50
8	Egypt	8	133	16.63
9	Iran	8	231	28.88
10	Saudi Arabia	7	191	27.29

### 2.7 Scientometric analysis of the organization

In order to understand which institutions have the most prominent contribution to the field of RES and cognitive function research, this study analyzed the number of documents issued by 443 institutions. First, visualization was performed by VOSviewer, and the results are shown in Figure 2C. The larger the circular node indicates that the number of documents issued is more; the node connection represents the association strength, and the thicker the connection indicates that the more times the two countries cooperate to issue documents; the node color represents different clusters.

Further analysis of the high‐productivity institutions in this field, Table 4 presents the institutions in the top 10 of the number of documents issued. According to the data in Table 4, Newcastle University from the United Kingdom published a total of 14 papers, accounting for 4.88% of the total number of documents issued in this field, and obtained a total of 500 citations. This was followed by Barcelona University from Spain, which issued a total of 12 articles and obtained 678 citations. The third place is Zhengzhou University from China, which issued a total of 8 articles and obtained a total of 220 citations. The institution with the most average paper citation times was Idibaps, 5 papers received 365 citations, and the average paper citation times reached 73 times.

**TABLE 4:** Top 10 institutions in the RES and cognitive function research field.

Rank	Institutions	Documents	Citations	Average Citation/Publication
1	Univ Newcastle	14	500	35.71
2	Univ Barcelona	12	678	56.50
3	Zhengzhou Univ	8	220	27.50
4	Anhui Med Univ	7	116	16.57
5	Idibaps	5	365	73.00
6	Fudan Univ	5	54	10.80
7	Chang Gung Univ	5	163	32.60
8	Hebei Med Univ	5	158	31.60
9	Panjab Univ	5	236	47.20
10	Qingdao Univ	5	25	5.00

### 2.8 Literature and reference analysis

The 10 most cited articles on RES in cognitive function studies are shown in Table 5. The most cited article is mainly to do clinical research and to investigate the effects of RES on memory performance in healthy older adults (Effects of Resveratrol on Memory Performance, Hippocampal Functional Connectivity, and Glucose Metabolism in Healthy Older Adults). The second most cited article is basic research that found that RES significantly improved memory deficits in high‐fat diet (HFD)‐fed mice (Resveratrol Attenuates Obesity‐Associated Peripheral and Central Inflammation and Improves Memory Deficit in Fed Mice a High‐Fat Diet). The third most cited article focused on RES preventing the increase in acetylcholinesterase activity and thereby preventing memory impairment in streptozotocin‐induced diabetic rats (Resveratrol prevents memory deficits and the increase in acetylcholinesterase activity in streptozotocin‐induced diabetic rats).

**TABLE 5:** The top 10 most‐cited articles in the RES and cognitive function research field.

Rank	Cited	Journal			Impact Factor (2021)	Author
		Year	Name	Country		
1	273	2014	*Journal of Neuroscience*	USA	6.709	A. Veronica Witte
2	246	2012	*Diabetes*	USA	9.305	Byeong Tak Jeon
3	178	2009	*European Journal of Pharmacology*	Netherlands	5.195	Roberta Schmatz
4	170	2013	*Age*	USA	4.648	David Porquet
5	160	2012	*Neurobiology of Aging*	England	5.133	Jaewon Chang
6	125	2014	*Behavioral Brain Research*	Netherlands	3.352	Laura L. Hurley
7	122	2014	*American Journal of Physiology‐Heart And Circulatory Physiology*	USA	5.125	Peter Toth
8	120	2013	*Biochemical And Biophysical Research Communications*	USA	3.322	Yong‐Na Zhao
9	110	2013	*Journal of Hypertension*	USA	4.776	Rachel H.X. Wong
10	108	2013	*Molecular Neurobiology*	USA	6.686	Rudimar L. Frozza

### 2.9 Generation and analysis of keyword co‐occurrence network

According to scientometric theory, keywords indicate hotspots and trends in the research field. Keyword analysis also provides a typical overview of research trends representing journals as they reflect the focus of the article or author. A co‐occurrence network view of keywords was drawn using VOSviewer for 287 articles, and keywords with a frequency greater than or equal to 5 among them were selected for visualization, and the results are shown in Figure 2D. The larger the circular node, the more times the keywords appear, and the more they represent the domain hotspots; the node connection represents the association strength, and the thicker the connection indicates that the two co‐appear in the same literature more times; the node color represents different clusters, that is the research theme. As shown in Figure 2D, the largest node is RES and cognitive function is the same as the research topic, and “RES” versus “cognition” is a cross‐keyword in these clusters, suggesting that RES may affect cognition. In fact, RES has been used clinically to improve cognition, and it has also been repeatedly verified in basic experiments that RES can improve cognition. This article will focus on the effects of RES on cognitive function. From Figure 2D, it can be seen that depression, AD, cerebral ischemia, diabetic cognitive impairment, and cognitive function in the elderly are all in the keywords of the co‐occurrence network. At the same time, we read through the abstract and full text of 287 articles and found that RES improved cognitive impairment mainly in young adults, the elderly and post‐menopausal women in basic and clinical research. The related disease is mainly AD, type 2 diabetes cognitive impairment (T2DM), mental illness, post‐stroke cognitive disorder, and neonatal hypoxic–ischemic injury. We, therefore, review in depth the effects of RES on cognitive impairment, and the study flow diagram of RES on cognitive function is shown in Figure 1.

## 3 HEALTHY YOUNG ADULTS

Enormous studies have examined how RES treats metabolic and neurodegenerative illnesses in animal and human models. However, the specific effects of RES on cell, tissue and organ function in healthy individuals are still unknown. According to a population‐based study, the high‐calorie diet is related to an increased risk of mild cognitive dysfunction^21^ or dementia in the elderly.^22^ Notably, caloric restriction has been indicated to extend the lifespan and improve learning as well as memory functions.^23^, ^24^ RES improved learning and memory function in normal C57BL/6J mice fed with an HC (high calorie) diet. It worked by the upregulation of multiple tumor suppressor 1 (p16) or downregulation of peroxisome proliferator‐activated receptor gamma (PPARγ) in the hippocampal CA1 region of mice to play the neuroprotective role.^12^ However, another study of RES effect on neural progenitor cells has shown that RES adversely affects hippocampal neurogenesis and cognitive function by activating AMP‐activated protein kinase (AMPK) and suppressing cyclic AMP response element‐binding protein (CREB) and brain‐derived neurotrophic factor (BDNF) signaling.^25^

**FIGURE 3:**
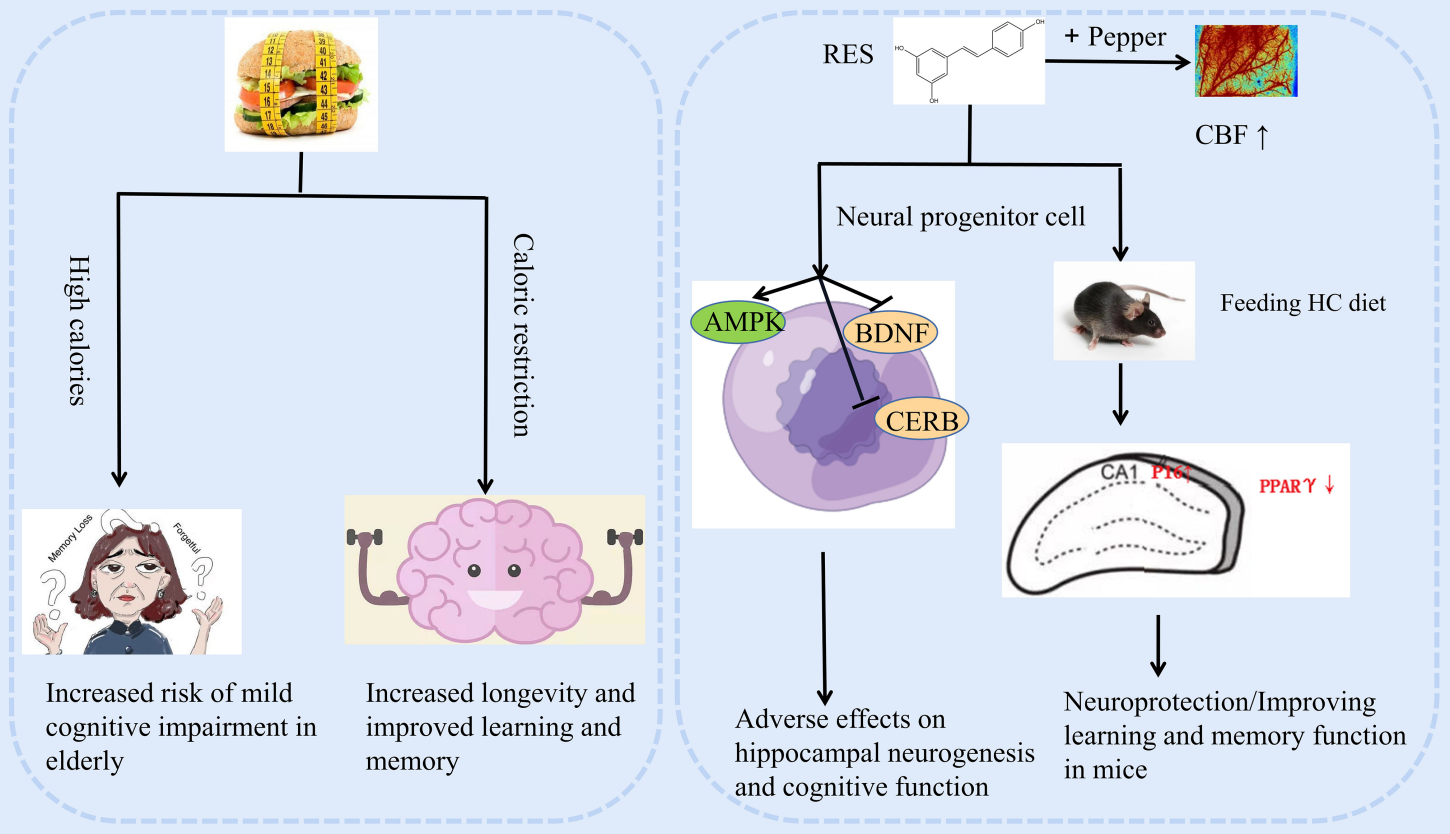
Scheme illustrating the role of RES on cognitive function in healthy young adults.

Blood‐borne brain metabolic substrates like oxygen and glucose have been shown to improve cognitive function in young healthy individuals. The combination of RES and pepper, in which the pepper increases the biological utilization of RES in the human body, could increase cerebral blood flow (CBF) without any significant changes in cognitive functions (Figure 3).^26^

## 4 HEALTHY OLDER ADULTS

As we all know, aging and cognitive dysfunction are closely related because aging impairs emotional and spatial learning memory. According to the most recent World Health Organization (WHO) statistics, there are expected to be 55 million people living with dementia in 2019 and 139 million people globally by 2050.^27^

**FIGURE 4:**
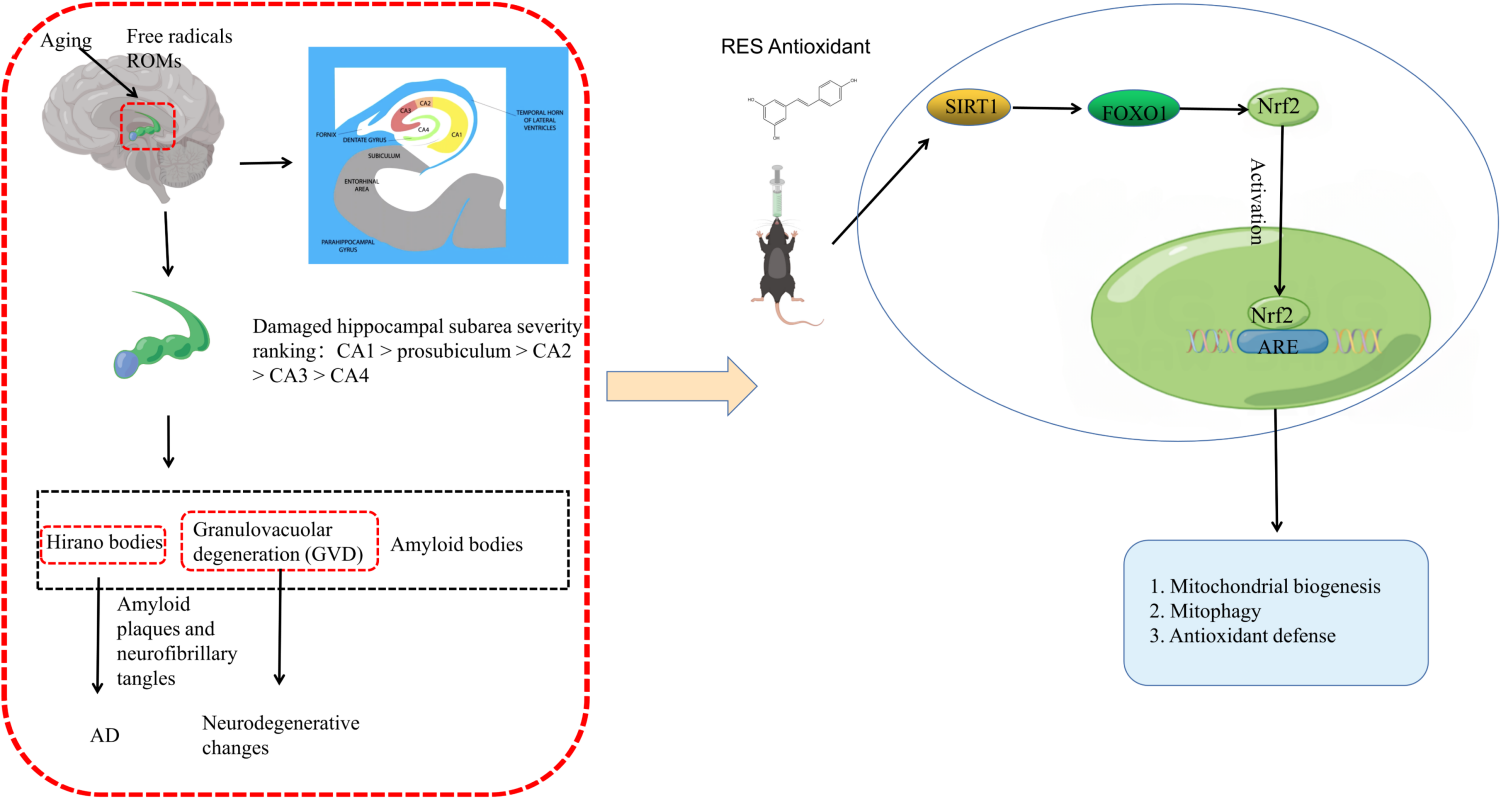
Scheme illustrating the role of RES on cognitive function in healthy older adults by figdraw.

The hippocampus in the aging human brain undergoes microscopic alterations, such as the development of amyloid bodies, granulovacuolar degeneration (GVD), and hirano bodies. Also, the two most well‐known pathology indicators of AD, amyloid plaques and neurofibrillary tangles, are observed in those who do not have dementia.^28^ GVD is a neurodegenerative alteration frequently seen in normal aging after 60 years old, predominantly in the hippocampal formation. The most seriously damaged neurons are those in the hippocampal CA1 area, followed by those in the presubiculum, CA2, CA3, and CA4 areas, in decreasing order of severity. In addition, some data indicated that free radicals play an important role in aging. The theory of oxidative stress, which is related to the reactions of biomolecules with oxides and peroxides, is based on free radicals, or reactive oxygen metabolites (ROMs). They cause many pathological degenerative changes that are related to aging (Figure 4).^29^, ^30^

When the body faces oxidative stress, reactive oxygen species (ROS) are metabolic byproducts essential for physiological function and can become toxic at high levels.^31^ Throughout the people's lifespan, these oxidative stressors gradually rise, affecting mitochondrial function and damaging all parts of the body, especially the central nervous system. Emerging evidence points to accumulated oxidative stress could be one of the major factors contributing to cognitive aging. RES acts as a natural antioxidant, as well as it can also delay aging and prevent cancer. However, it has not yet been determined how RES affects learning and memory in normal aging models.

90 days of RES treatment for sedentary, overweight older adults showed a considerable increase in psychomotor speed but had no discernible effects on other cognitive function areas.^32^ In another clinical study, a significant impact of RES on the retention of words over 30 minutes was observed, along with enhanced glucose metabolism and increased hippocampal functional connectivity.^33^ For 15‐month‐old mice, RES combined with dimethyl fumarate and methylene blue improved short‐term and long‐term memory. The compounds are shown to enhance antioxidant defense, mitochondrial biogenesis, and mitophagy in the hippocampus by activating the Nrf2/ARE pathway, which lowers the level of oxidative damage to mtDNA (Figure 4).^34^ Age‐related learning and memory impairment is reversed by RES. The findings of this study imply that RES helps prevent cognitive deficits in aged rats by limiting the production of inflammatory cytokines.^35^ Besides, RES has been shown to protect against age‐related cognitive impairment, especially when administered over a long period (10 mg/kg for 8 months of treatment).^36^

## 5 POST‐MENOPAUSAL WOMEN

**FIGURE 5:**
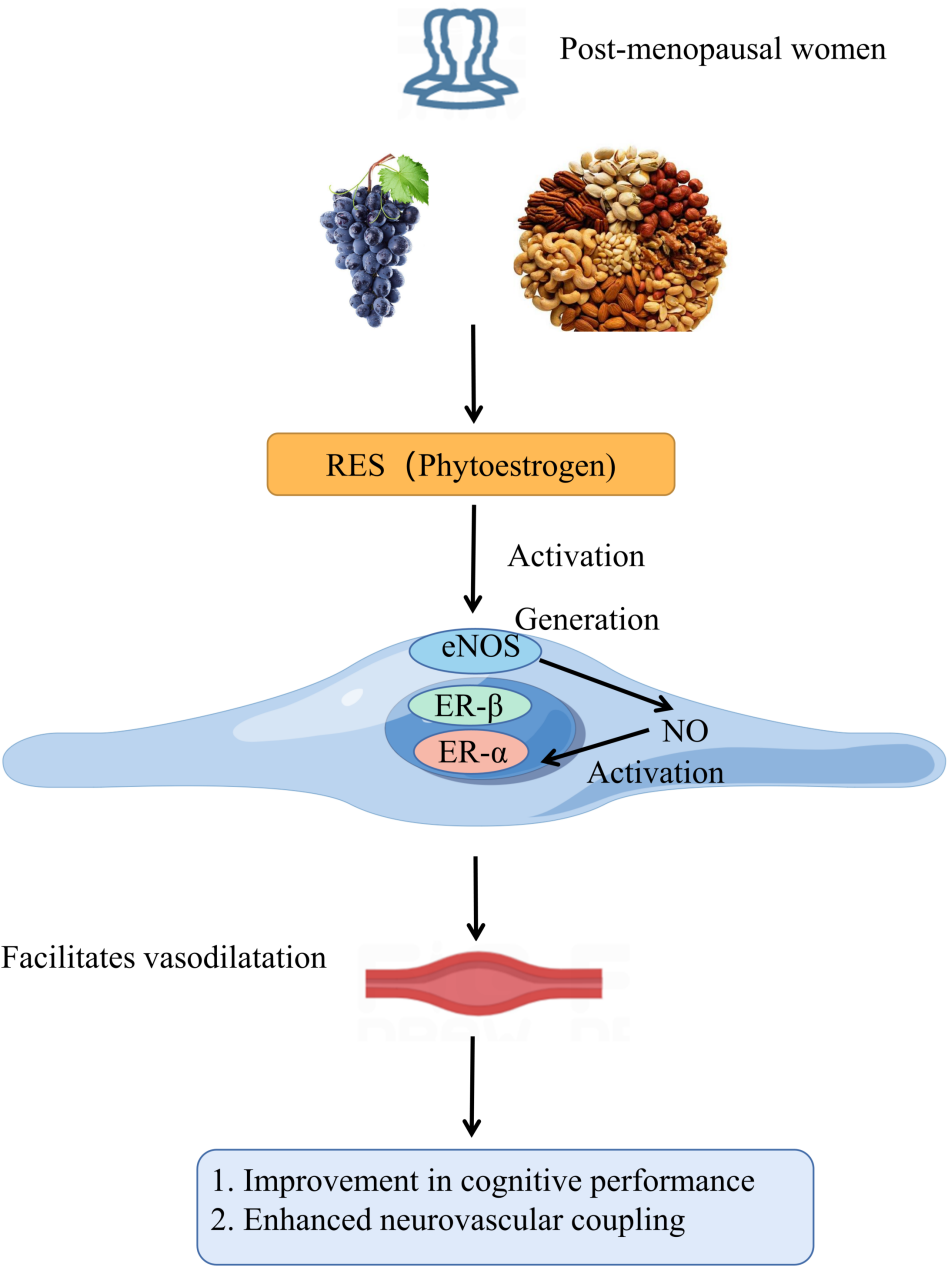
Scheme illustrating the role of RES on cognitive function in post‐menopausal women by figdraw.

By raising endothelial nitric oxide (NO), estrogen activates the estrogen receptors (ER) on endothelial cells to promote vasodilation (Figure 5).^37^ Compared to men or younger women, women over 55 have a higher risk of cardiovascular diseases.^38^ Thus, these differences can be partially attributable to the rapid loss of estrogen in circulation, which could hasten age‐related arterial stiffening and decrease tissue perfusion by reducing endothelium‐dependent vasodilatation. Undoubtedly, estrogen is crucial for maintaining memory, regulating metabolism, and maintaining bone health in women. According to a meta‐analysis, post‐menopausal women perform worse than pre‐menopausal women on verbal memory and executive function tests.^39^ Therefore, the loss of estrogen may increase the risk of cardiovascular and cerebrovascular diseases resulting in impaired cerebral perfusion, which is connected to accelerated cognitive decline.

It has been demonstrated that RES, a phytoestrogen present in various foods like grapes, berries and nuts, improves the function of endothelium vasodilators in humans through multiple mechanisms, including activating endothelial ER and consequently enhancing NO production.^40^ In a clinical experiment of chronic RES administration (for 14‐week treatment) for post‐menopausal women, an improvement in cognitive function was observed, accompanied with enhanced neurovascular coupling.^41^ A longer‐term study that reached 12 months of RES treatment showed the same clinical effect, including improved overall cognitive performance and decreased cerebrovascular response to cognitive stimuli.^42^, ^43^

Animal experiments have better revealed the mechanism of RES to protect cognitive function. It is indicated that RES worked not only by decreasing oxidant stress indices, maintaining the structure of mitochondria, and decreasing condensed chromatin in the pyramidal cells of the hippocampal CA1 region but also by avoiding the effects on the uterus.^44^

Long‐term supplementation with low‐dose RES tended to improve post‐menopausal women's cognition and cerebrovascular function, which may mean slowing the accelerated cognitive deterioration due to aging and menopause, particularly in late‐life women. Further, explore of RES is warranted to determine whether these cognitive benefits can lower the risk of cognitive impairment.

## 6 RES AND ALZHEIMER'S DISEASE

AD is a chronic neurodegenerative brain condition that steadily erodes memory and cognitive abilities. For elderly adults, it is the most typical cause of dementia.^27^ The most important hallmark of AD is the presence of amyloid beta (Aβ) peptide as well as neurofibrillary tangles resulting from tau hyperphosphorylation in the brain, which indicates that they may be the primary trigger for neuronal damage.^45^, ^46^ Until now, there are only six drugs for the treatment of AD certified by the U.S. Food and Drug Administration (FDA). Not only that, most drugs can only relieve symptoms and cannot slow the progress of the disease.^47^ Clinical treatment of AD is facing enormous challenges.

In the last decades, RES has been found to be a potential protector compound for the treatment of neurodegenerative diseases (i.e., AD, Parkinson's disease, and amyotrophic lateral sclerosis) resulting from its antioxidant and anti‐inflammatory properties. Notably, RES can also modulate different molecular pathways in neurodegenerative diseases by specifically activating silent information regulator‐1 (SIRT1). The protein deacetylase SIRT1 is the mammalian homolog of the yeast silent information regulator‐2 (SIRT2), a member of the sirtuin family that has received considerable interest as a potential mediator of lifespan extension in a number of model animals.^48^

**FIGURE 6:**
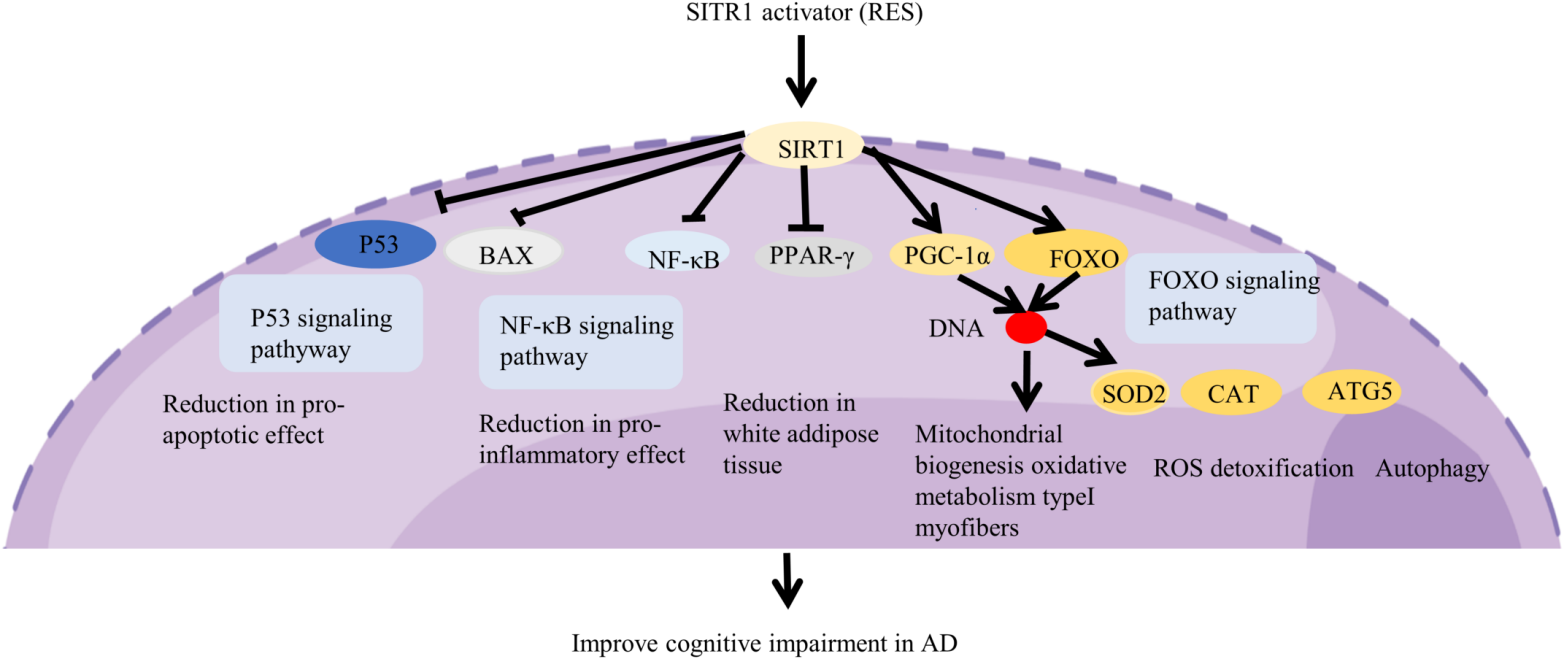
Scheme illustrating the role of RES on cognitive impairment in AD by figdraw.

In the clinical experiment of AD patients, RES reduces Aβ levels, improves brain volume, reduces the Mini‐mental status score, and improves AD scores.^49^, ^50^, ^51^ Furthermore, it is indicated that physiological doses of RES are safe and well tolerated.^52^ In addition to being clinically useful in treating AD, RES in combination with donepezil hydrochloride has also been demonstrated to be effective in enhancing inflammatory factor indicators, fostering cognitive function, and facilitating patient prognosis.^53^ Similarly, RES combined with rifampicin significantly improved cognition, reduced Aβ peptide accumulation, and recovered synaptophysin levels in the hippocampus of mice. It is indicated that RES enhanced the levels of BDNF and its precursor, pro‐BDNF in the hippocampus (Figure 6).^54^

RES can activate the human silent information regulator 2/SIRT‐1 and inhibit the nuclear factor‐κB, 5‐lipoxygenase and cyclooxygenase‐2 (COX‐2) to reduce the proinflammation pathways. It is also associated with increased levels of interleukin (IL)‐10, interferon‐γ and IL‐17 are also decreased.^20^ Long‐term administration of RES tends to get a better curative effect and prevent dementia, whether for AD transgenic (3xTg‐AD) mice or non‐transgenic (No‐Tg) mice. Mice were fed with a diet supplemented with 100 mg/kg of RES from 2 months of age during the 10 months.^55^ Hippocampal injection of Aβ1‐42 induced AD models in rats and mice have also been used to investigate the effect of RES.^56^, ^57^ The prevention of neurodegenerative illnesses, maintenance of these conditions, and facilitation of their recovery are all closely linked to anti‐inflammatory and antioxidant activities.

## 7 RES AND DIABETES‐RELATED COGNITIVE IMPAIRMENT

As one of the most common and important complications of diabetes, diabetes‐related cognitive dysfunction seriously affects the quality of patients' life. Besides, more and more clinical and epidemiological data have shown that the incidence of cognitive impairment in T2DM patients is higher than that in non‐diabetic patients.^58^ The pathogenesis of diabetes‐related cognitive impairment is complex and unclear, and current studies have found that the possible mechanisms by which T2DM increases the risk of cognitive impairment mainly include insulin resistance, neuroinflammation, and brain neuronal injury.^59^

**FIGURE 7:**
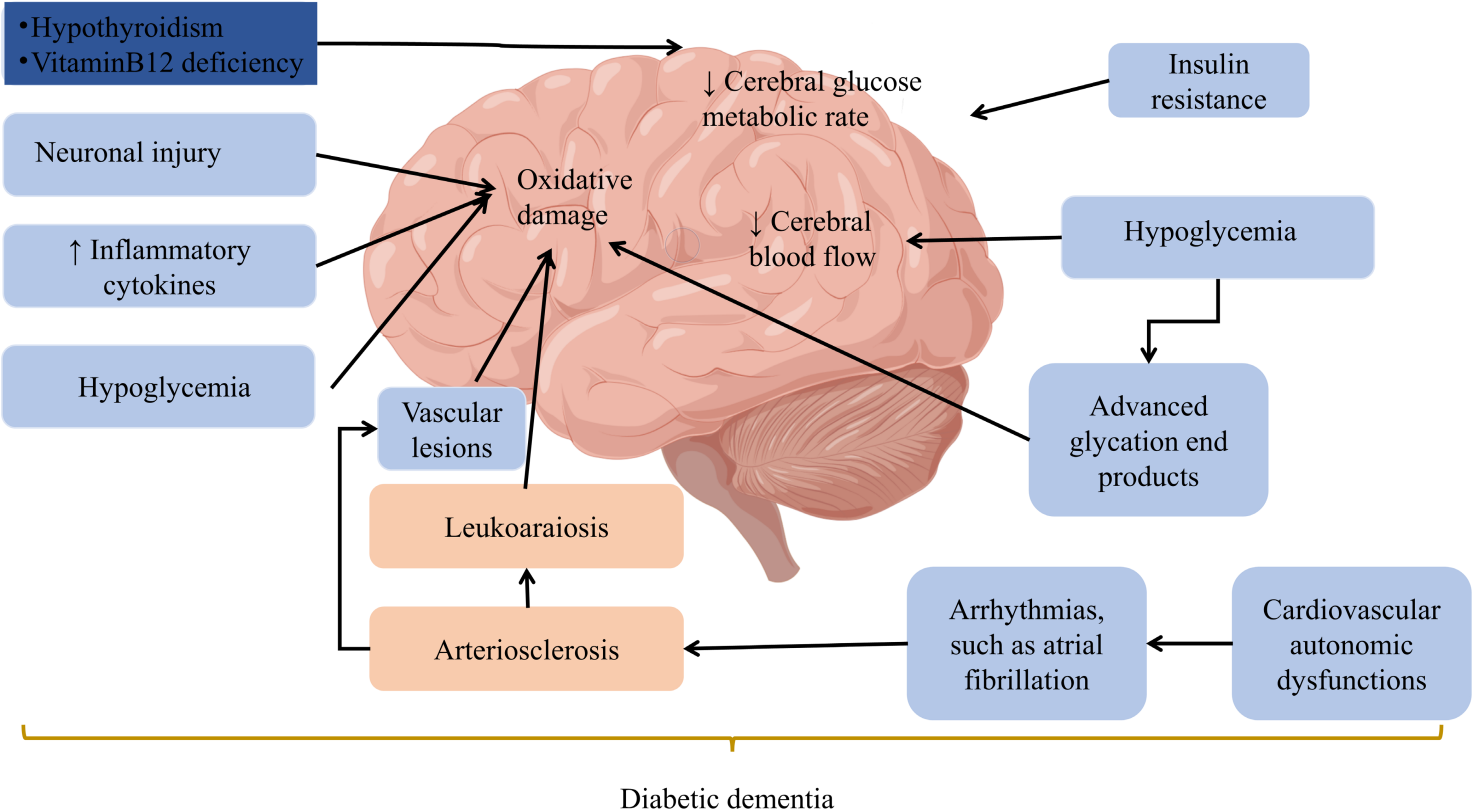
Presumed causes of memory impairment in patients with diabetes by figdraw.

Experimental studies have found that the development of diabetes‐related cognitive impairment is closely related to SIRT1, one of the modulators of insulin resistance that plays an important role in learning and memory.^60^ Interestingly, RES is considered to be a natural activator of SIRT1. Furthermore, it has been found to be effective in improving learning and memory ability without changing SIRT1 expression levels,^61^ which indicates that the effect of RES on cognitive improvement may be partially dependent on activating SIRT1. In addition to SIRT1, the damage of synapses in the brain structure of diabetic patients is also one of the reasons for diabetes‐related cognitive decline (Figure 7). RES has been observed to significantly enhance learning ability in streptozotocin (STZ)‐induced diabetic rats, mainly due to its antioxidant and anti‐inflammatory activities, ultimately promoting synaptic plasticity in the hippocampal region.^62^, ^63^, ^64^ Synaptic plasticity includes modifications in the structure and function of synapses, which are integral components of learning and memory.^65^ Meanwhile, cognitive decline is associated with a gradual decrease in structural and functional plasticity in brain regions that play a crucial role in cognitive function.^66^ These expand plasticity under physiological and pathological conditions in the brain in terms of molecular mechanisms by which RES improves cognitive function.

Clinical studies revealed that one potential mechanism of T2DM‐related cognitive impairment could be chronic hypoperfusion of the brain, possibly due to progressive microvascular dysfunction associated with advanced glycation end products (AGEs) formation in T2DM.^67^ Moreover, it has been shown that adequate CBF is crucial for brain function.^68^ Interestingly, RES was found to be effective in enhancing vasodilation in two randomized controlled trials, thereby reducing cognitive impairment in patients with T2DM.^69^, ^70^ Besides, it was further explored that a single dose of 75 mg RES could significantly increase neurovascular coupling capacity, with the greatest improvement observed using the lowest dose.

In summary, the improvement of cognitive dysfunction by RES in diabetes has been studied in both animals and patients. RES improved cognitive dysfunction mainly by reducing oxidative stress and inhibiting synaptic loss. On the other hand, clinical studies indicated the effect of RES on CBF, then reducing cognitive impairment in T2DM patients.

## 8 RES AND MENTAL ILLNESS

Depression is one of the most common neuropsychiatric disorders, and schizophrenia is a serious mental disorder, both of which are associated with cognitive impairment.^71^ As we all know, stress is one of the important causes leading to the development of depression. The chronic unpredictable mild stress (CUMS) procedure is a classic animal model used to reveal depression‐like behavior and cognitive deficits in rodents.^72^ In numerous animal models, it has been found that depression is closely associated with reduced hippocampal neurogenesis, altered synaptic morphology, and decreased BDNF expression.^73^ BDNF, a crucial member of the neurotrophin family, is broadly distributed in the cerebral cortex and hippocampus.^74^ BDNF is beneficial to the survival of existing neurons, promotes the growth and differentiation of neurons and synapses,^75^ and also promotes long‐term memory formation.^76^

**FIGURE 8:**
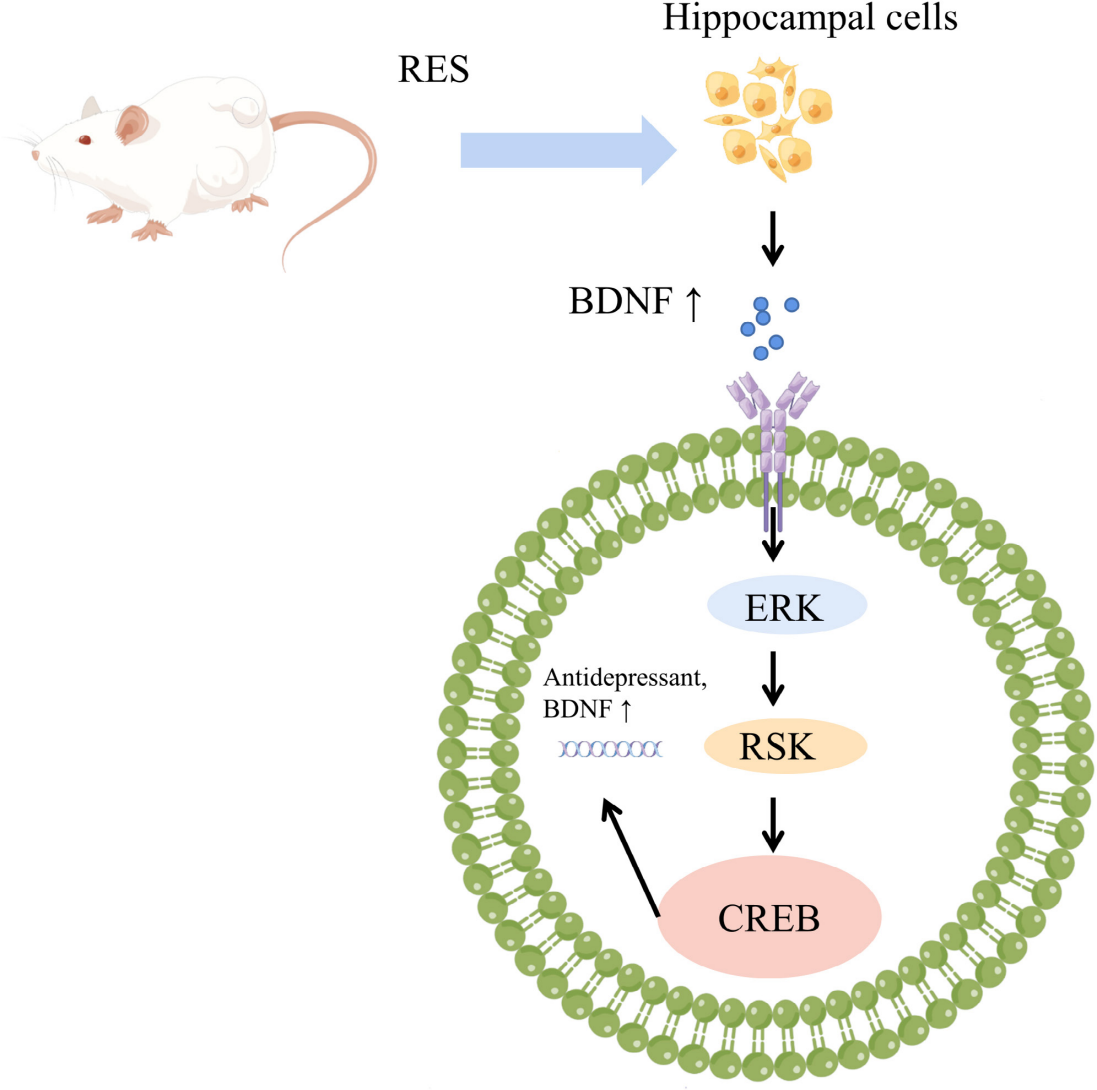
Scheme illustrating the role of RES on cognitive impairment in mental illness by governing the ERK‐CREB signaling pathway by figdraw.

It has been indicated that RES improved cognitive impairment by up‐regulating BDNF expression in the rat hippocampus.^77^ Additional research showed that the ERK‐CREB signaling pathway, which is linked to learning, memory, and neuroplasticity and essential for controlling various brain activities, is strongly tied to BDNF production.^78^ According to experimental research, the enhanced BDNF expression via the ERK‐CREB pathway in the hippocampus of stressed rats may be the cause of the neuroprotective action of RES (Figure 8).^79^ In addition to BDNF, SIRT1 is also essential for maintaining cognitive function,^80^ and RES can reverse depression‐like behavior by activating SIRT1.^81^ Clinical studies have found that RES supplementation for 1 month (200 mg/day) did not improve episodic memory, working memory, attention and concentration ability, inhibitory control, interference measures, selective attention and mental flexibility in patients with schizophrenia compared with placebo.^82^ RES treatment on cognitive demand tasks did not significantly improve cognitive function in schizophrenia clinical studies. It has been discovered that RES's limited bioavailability and diminished effectiveness in vivo may be the cause of the absence of cognitive benefits.^83^ There are several variables that affect the bioavailability of polyphenols, including food composition, dietary habits, and the pathophysiological and nutritional circumstances of a person.^84^ There are many reasons why RES lacks efficacy in human studies, which need to be further explored by researchers.

## 9 RES AND POST‐STROKE COGNITIVE DISORDER

**FIGURE 9:**
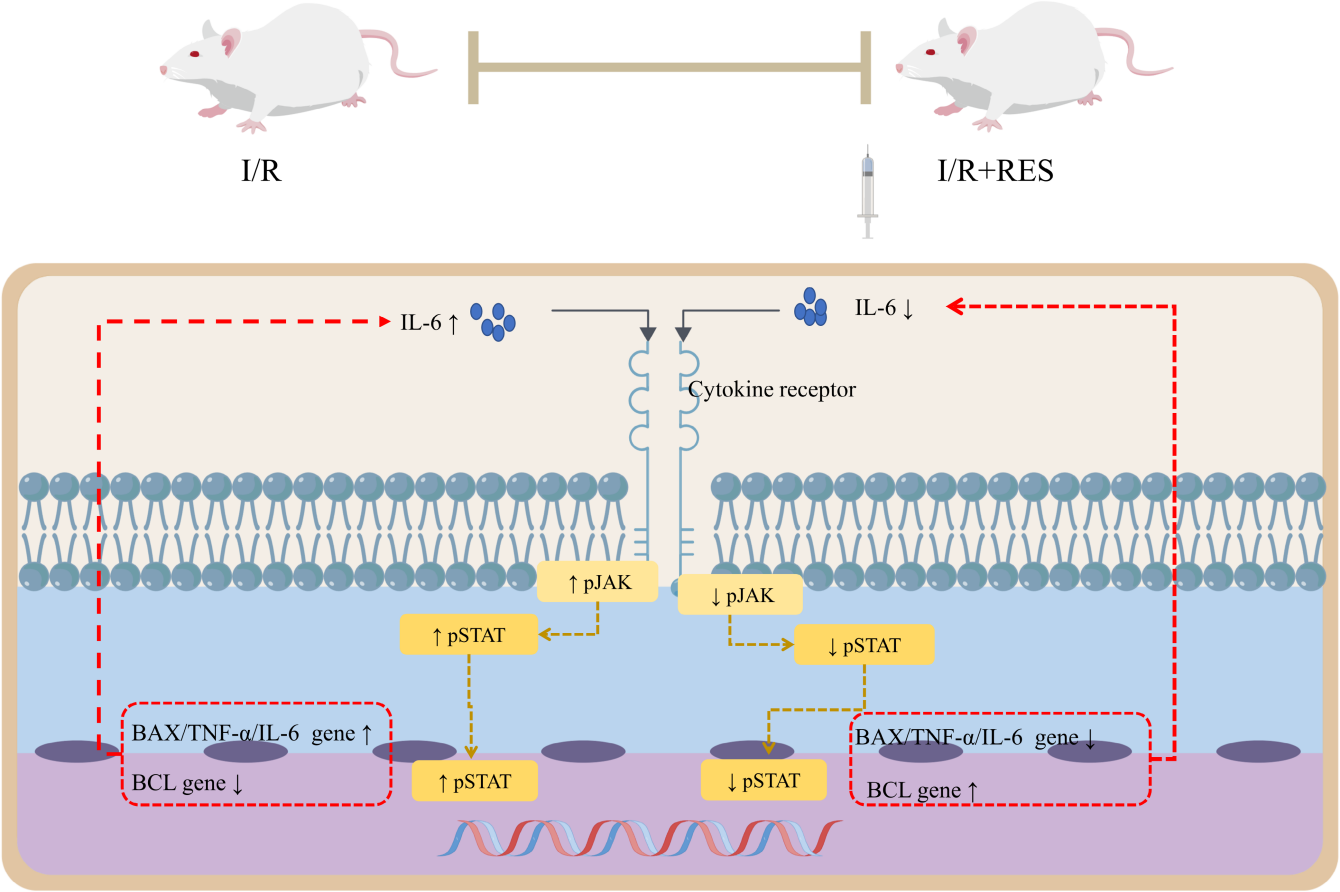
Scheme illustrating the role of RES on cognitive impairment in post‐stroke cognitive disorder by governing the JAK/ERK/STAT signaling pathway.

Stroke is an acute cerebrovascular disease caused by cerebral blood supply disorders, which is one of the top three causes of death in the population of developed countries.^85^ Epidemiological surveys have shown that the annual number of stroke patients in China is more than 1.5 million, with the disability rate reaching 86.5%. Ischemic stroke is the most common type of stroke, accounting for about 60%–80% of the total number of strokes. Stroke is closely related to the development and deterioration of cognitive impairment. Animal studies have found that cognitive impairment caused by stroke is closely associated with neuronal apoptosis in the hippocampus,^86^ and oxidative stress has the greatest effect on hippocampal damage, thus leading to memory impairment.^87^ It has been shown that RES effectively reduced inflammatory response and oxidative stress by regulating the activation of JAK/ERK/STAT signaling pathway, and then improved hippocampal neuron loss and cognitive impairment in ischemia‐reperfusion (I/R) rat models (Figure 9).^88^ While Piceatannol, a natural hydroxylated analogue of RES,^89^ is indicated to improve cognitive dysfunction by maintaining the balance of antioxidant/oxidative system and protecting hippocampal neurons from apoptosis, in addition, these effects are involved by SIRT1/FoxO1 signaling pathway,^90^ which also involves ERK‐CREB signaling pathway.^91^

At present, the mechanism of RES on cognitive impairment caused by stroke is mainly related to anti‐inflammatory, antioxidant and anti‐apoptotic, and ultimately improves hippocampal neuronal loss and cognitive impairment. However, the studies of RES on cognitive impairment in stroke mainly focus on in vitro and in vivo models, and there is no related clinical research.

## 10 RES AND NEONATAL HYPOXIC–ISCHEMIC INJURY

**FIGURE. 10:**
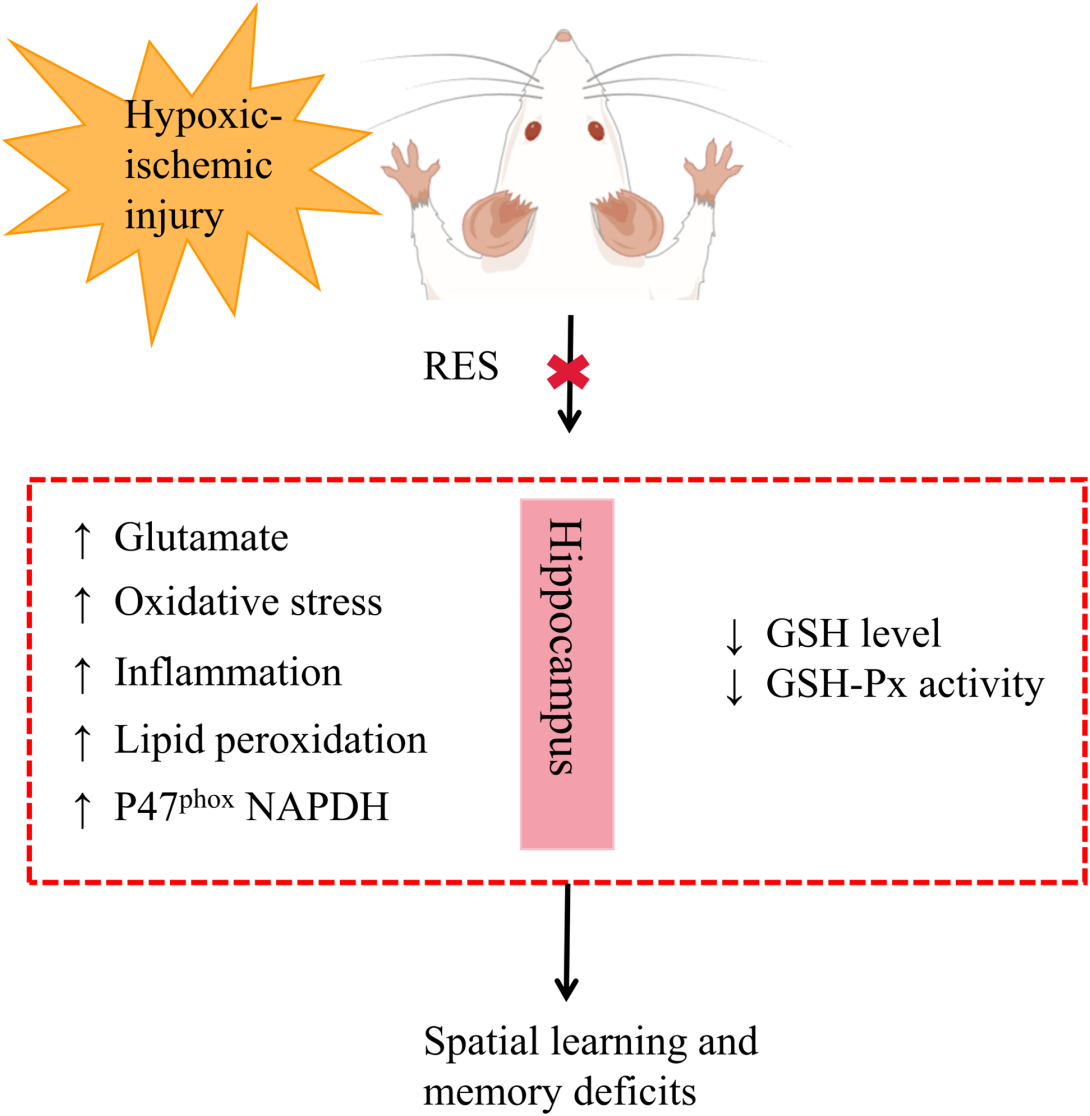
Scheme illustrating the role of RES on cognitive impairment in neonatal HI injury by figdraw.

Neonatal hypoxic–ischemic (HI) injury causes brain dysfunction in children, such as permanent limb injury, cerebral palsy, epilepsy, and cognitive impairment.^92^ Compared with adults, immature neonatal brains are more susceptible to oxidative stress. Previous studies have found that thiobarbituric acid‐reactive substance (TBARS), a marker of lipid peroxidation, increased in the hippocampus of HI models compared with the normal group. Lipid peroxidation in the hippocampus is mainly due to increased ROS during hypoxia, which in turn leads to neuronal cell membrane damage,^93^, ^94^, ^95^ suggesting that oxidative stress is one of the potential triggers of HI behavioral disorders. In addition, clinical studies have found that HI injury is mainly characterized by neuroinflammation, and anti‐inflammatory activity has been observed to improve brain damage after HI.^96^ As we all know, RES is effective in inhibiting nitric oxide production and increasing the activity of antioxidant enzymes (e.g., glutathione (GSH), superoxide dismutase (SOD), glutathione peroxidase (GSH‐Px), and catalase) during hypoxia (Figure 10).^97^, ^98^, ^99^ RES could induce the expression of mitochondrial enzymes and reduce the ability of mitochondria to oxidative stress and injury,^100^ which improved HI cognitive impairment.

Furthermore, RES dose‐dependently reduced the HI‐induced rise in nicotinamide adenine dinucleotide phosphate (NADPH) oxidase gene expression in the hippocampus,^101^, ^102^ and RES significantly decreased 8‐OHdG (a specific biomarker of oxidative DNA damage) levels in the serum and brain of HI rat models.^103^ It is also indicated that RES could produce antioxidant effects under ischemic stress by regulating the SIRT1‐PGC‐1α signaling pathway.^104^ While pterostilbene (PTE), a methylated derivative of RES, could inhibit oxidative stress, programmed cell death, inflammation and brain injury in neonatal rats by regulating the HO‐1 signaling pathway to improve cognitive impairment.^105^ In addition, the timing of RES administration has been investigated by numerous investigators. Prior to HI, early supplementation with RES was effective in improving long‐term cognitive impairment due to HI,^106^ and maternal RES supplementation reversed sensorimotor and cognitive deficits caused by HI, and RES affected brain metabolism in addition to protecting nerves by antioxidant properties and inhibiting apoptosis. Generally, RES is a potential therapeutic agent that can restore HI cognitive dysfunction through anti‐oxidation and anti‐inflammation through multiple signaling pathways. However, RES on HI cognitive impairment studies have not been clinically applied and are limited to animal studies.

## 11 CONCLUSIONS AND FUTURE PERSPECTIVES

In order to find new research hotspots, this work suggests a novel approach to investigate the enhancement of cognitive performance by RES. Based on the findings of scientometric research, a thorough and in‐depth review was carried out. This article reviews the therapeutic effects of RES on cognitive function and provides a novel and unique perspective for its treatment.

It is important to elucidate the regulatory mechanisms of RES on cognitive impairment, including healthy adults, healthy older adults, post‐menopausal women, AD, diabetes‐related cognitive dysfunction, mental illness, post‐stroke cognitive disorder, neonatal HI injury accompanied by deterioration of neuronal injury‐related signaling pathways and factors implicated. The profile of intricate signaling pathways in many diseases can help us better understand the role that RES plays in various diseases. It is worth noting that the mechanism of action of RES involves numerous different diseases, including Nrf2/ARE signaling pathway in the elderly, ERK/CREB/BDNF signaling pathway in mental illness, JAK/ERK/STAT signaling pathway and Sirt1/FoxO1 signaling pathway in post‐stroke cognitive disorder, and SIRT1‐PGC‐1α and HO‐1 signaling pathways in HI injury, which are closely related to cognitive dysfunction. Therefore, the ability of RES to target different underlying mechanisms highlights its potential to treat a wide range of cognitive impairments.

Although the benefits of RES for improving cognitive function have been well documented in experimental animals, human clinical trials are only beginning to be explored.^107^ Conflicting results exist regarding the effects of RES in humans, with some studies finding that RES improves cognitive function, but others finding that RES does not improve cognitive function.^33^, ^108^ RES (250 and 500 mg) injection caused dose‐dependent increases in cerebral blood flow, stimulating the frontal cortex during the execution of cognitive tasks, according to Kennedy et al.'s investigation into the vasodilatory effects of RES in 22 healthy people.^109^ However, cognitive function was not compromised. In a similar study, Wightman et al. conducted a randomized, double‐blind placebo‐controlled study of 60 healthy adults treated with placebo or RES for 28 days. Performance on cognitively demanding tasks did not significantly improve cognitive function at the end of study.^83^ Interestingly, Witte et al. studied 23 healthy, overweight older individuals who consumed 200 mg of RES daily for 26 weeks, and they offered early proof that RES supplementation enhanced memory function.^33^

Taken together, these studies support the hypothesis that chronic RES intake may positively affect brain function. Possibly, beneficial effects on the brain may translate into behavioral improvements following RES ingestion for sufficiently long periods of time. But why does RES lack efficacy in some human studies? First, the optimal dose, timing, and duration of RES are unknown. Second, RES has relatively low bioavailability due to its massive and rapid hepatic metabolism. Therefore, it has become challenging to determine the optimal dose, time and duration of RES and improve the bioavailability of RES, which many researchers need to overcome.

## AUTHOR CONTRIBUTIONS

Wenling Tu: Conceptualization, Writing‐original draft, Preparation. Meiying Song: Conceptualization, Writing‐original draft. Xiang Fan: Conceptualization, Supervision, Writing‐review & editing. All authors read and approved the final manuscript. All data were generated in‐house, and no paper mill was used. All authors agree to be accountable for all aspects of work ensuring integrity and accuracy.

## CONFLICT OF INTEREST STATEMENT

All authors declare there is no potential conflicts of interest include employment, consultancies, stock ownership, honoraria, paid expert testimony, patent applications/registrations, and grants or other funding.

## Data Availability

The data that support the findings of this study are available from the corresponding author upon reasonable request.
